# Clinical features and outcomes of nine children with acute necrotizing encephalopathy

**DOI:** 10.3389/fped.2025.1615960

**Published:** 2025-08-06

**Authors:** Yahua Zhang, Lei He, Jingran Xu, Piaosi Wang, Hehe Chen

**Affiliations:** ^1^ Department of Infectious Diseases, Women and Children’s Hospital of Ningbo University, Ningbo, Zhejiang, China; ^2^ Department of Critical Care Medicine, Women and Children’s Hospital of Ningbo University, Ningbo, Zhejiang, China

**Keywords:** children, acute necrotizing encephalopathy, influenza virus, prognosis, clinical analysis

## Abstract

**Objective:**

To report the clinical features, cranial imaging findings, treatment approaches and outcomes of pediatric acute necrotizing encephalopathy (ANE) to improve early diagnosis and treatment strategies of this rare but severe condition.

**Methods:**

Retrospective analysis of nine children with ANE, admitted to the Pediatric Intensive Care Unit (PICU) of Women's and Children's Hospital of Ningbo University (2019–2024) was performed. Clinical presentations, laboratory results, neuroimaging results, treatment modalities, and outcomes were retrospectively evaluated. Survivors were followed up and their function evaluated using the Pediatric Overall Performance Category scale.

**Results:**

Patients (age range 9 months to 14 years) predominantly presented with fever, seizure and altered consciousness. Influenza A was the most common antecedent infection. All cases progressed to symmetric multifocal lesions, with elevated inflammatory markers like interleukin-6 (IL-6). Brain magnetic resonance imaging (MRI) typically showed symmetric bilateral thalamic lesions. Acute necrotizing encephalopathy Severity Score (ANE-SS), a severity index based on neurological symptoms, shock, and brainstem involvement, was significantly lower in survivors than in non-survivors (*P* < 0.05). Survivors received early immunomodulatory treatments, including high-dose methylprednisolone, intravenous immunoglobulin (IVIG), and plasma exchange (PLEX). The overall mortality rate was 56%. Survivors showed significant neurological improvement after rehabilitation therapy.

**Conclusion:**

ANE occurs commonly after influenza. Yet, many many children have influenza, and not many have ANE.It is typically presents with bilateral thalamic lesions and systemic inflammation.Hyperpyrexia and inflammatory markers are valuable prognostically indicators, and ANE-SS accurately predicts mortality risk. Early combined immunomodulatory therapy and rehabilitation may improve outcomes. These findings contribute to the understanding of clinical and imaging predictors and allow early identification, prognostication, and individualized management.

## 1 Introduction

Acute Necrotizing Encephalopathy (ANE) is a rare but aggressive and potentially fatal neurological disorder, typically caused by viral infections. Clinically, ANE presents with sudden onset of encephalopathy, including seizures and altered consciousness, and may be complicated by shock, multiple organ dysfunction syndrome (MODS), and disseminated intravascular coagulation (DIC) (1).

Originally reported by Mizuguchi et al. (2) in 1995, ANE is most frequently associated with influenza virus infections. Although the exact pathogenesis remains unclear, diagnosis is based on clinical presentation, laboratory findings, and characteristic neuroimaging (MRI), most prominently symmetric bilateral thalamic lesions (3). Early treatment with the intravenous corticosteroids, immunoglobulins, and plasma exchange (PLEX) has been proposed to improve outcomes. However, the optimal timing, dose, and combination of the treatments are undefined, and no treatment regimen has been applied worldwide (4, 5). Mortality rates are found to be 30%–70%, and fewer than 10% of survivors achieve full neurological recovery (6).

A retrospective review of the clinical characteristics, cranial imaging findings, treatment interventions, and outcomes of pediatric ANE cases from a single-center was conducted to enhance clinical recognition and management of this rare condition.

## 2 Materials and methods

### 2.1 Study subjects

This retrospective analysis included clinical data of nine children diagnosed with ANE who were admitted to the Pediatric Intensive Care Unit (PICU) of Women and Children's Hospital of Ningbo University from March 2019 to September 2024. Inclusion criteria were: (1) ANE diagnostic made according to Mizuguchi et al. (2)'s criteria; (2) Age between 29 days and 14 years; and (3) Complete clinical records available. Exclusion criteria were: (1) Acute encephalopathy caused by other etiologies; (2) Patients with incomplete clinical data. This study was approved by the Institutional Review Board (IRB) of Women's and Children's Hospital of Ningbo University (Approval No.: 2024ZL961). Written informed consent was obtained from guardians of the patients. Data of all patients were anonymized to protect privacy.

### 2.2 Methods

#### 2.2.1 Clinical data collection

Clinical data included demographics (sex and age), underlying diseases, clinical features, etiological examinations, inflammatory markers [C-reactive protein (CRP), procalcitonin (PCT), interleukin(IL)-6, and interleukin(IL)-10], liver enzymes [alanine aminotransferase (ALT), aspartate aminotransferase (AST)], lactate dehydrogenase (LDH), coagulation function [prothrombin time [PT], activated partial thromboplastin time [APTT], fibrinogen and D-dimer], cytokines, cerebrospinal fluid (CSF) protein examination, cranial imaging findings, and treatment regimens.

Clinical parameters were set according to widely accepted pediatric guidelines. Fever was >38.5°C, and hyperpyrexia was >40°C, consistent with the standard definition of pediatric fever. Shock was defined as age-adjusted hypotension and/or signs of poor perfusion, such as tachycardia, delayed capillary refill time (>2 s), and/or elevated serum lactate (>2 mmol/L). Acute liver injury was defined as ALT or AST >3 times the upper limit of normal. Acute kidney injury was defined as elevated serum creatinine or decreased estimated glomerular filtration rate (eGFR). Acute myocardial injury was defined as elevated troponin or abnormal findings on transthoracic echocardiography (TTE). Electrocardiogram (ECG) changes, such as ST-segment depression, T wave inversion, or conduction abnormalities, were also considered indicators of myocardial injury. All the laboratory parameters were interpreted using age-specific reference ranges. The interval between fever onset and the onset of seizure or altered consciousness was determined based on caregiver reports and medical records.

#### 2.2.2 Diagnostic criteria for pediatric acute necrotizing encephalopathy

ANE was diagnosed at PICU admission, typically within 24 h, based on initial clinical evaluation, laboratory findings, CSF protein examination, and neuroimaging studies. The diagnostic criteria were based on Mizuguchi et al. (2) and included the following findings: (1) Acute encephalopathy symptoms (e.g., seizures and altered consciousness) following febrile disease; (2) Elevated serum transaminases without hyperammonemia; (3) Normal CSF cell count accompanied by significantly elevated protein levels; (4) Symmetric, multifocal lesions involving bilateral thalami as identified by cranial Computed Tomography (CT) or MRI, with possible involvement of the periventricular white matter, internal capsule, putamen, brainstem, and cerebellum; (5) Exclusion of encephalopathy due to infectious, metabolic, toxic, mitochondrial, or autoimmune diseases. Early and accurate diagnosis facilitated timely intervention and prognostic assessment.

#### 2.2.3 Pathogen detection

Pathogens were identified by direct immunofluorescence assay, rapid influenza diagnostic test (RIDT), real-time reverse transcription polymerase chain reaction (RT-PCR), and metagenomic next-generation sequencing (mNGS).

#### 2.2.4 Acute necrotizing encephalopathy severity scale

Severity of disease was determined by the Acute Necrotizing Encephalopathy Severity Scale (ANE-SS), which was a prognostic indicator during the early stage of hospitalization. The ANE-SS was established at the time of PICU admission based on clinical judgment by evaluation, laboratory findings, and neuroimaging findings obtained within the first 24 h. The scoring system ranges from 0 to 9 points and includes the following components: (1) Shock (3 points): defined as persistent hypotension necessitating fluid resuscitation or vasopressor support, with age-adjusted hypotension thresholds and/or elevated serum lactate levels (>2 mmol/L), and clinical features such as tachycardia, delayed capillary refill, and altered mental status; (2) Brainstem lesions (2 points): determined by MRI or CT evidence of midbrain, pons, or medulla involvement, supported by neuroradiological evaluation, and associated with clinical findings such as abnormal pupillary reflexes or irregular respiration; (3) Age >48 months (2 points); 4. platelet count <100 × 10⁹ /L (1 point); 5. CSF protein >60 mg/dl (1 point). The patients were categorized as low risk (0–1 points), moderate risk (2–4 points), high risk (5–9 points). Higher ANE-SS scores were associated with worse neurologic outcomes (7).

#### 2.2.5 Prognosis and follow-up

Telephone follow-up was performed at 1, 3, and 6 months post-discharge in all surviving patients. In addition to telephone follow-up, serial cranial MRI scans were performed in surviving patients to monitor disease progression and recovery. Neurologic function was evaluated by employing the Pediatric Overall Performance Category (POPC) scale (8), which classifies functional status scores as follows: 1 point: Good; 2 points: Mild abnormalities; 3 points: Moderate abnormalities; 4 points: Severe abnormalities; 5 points: Coma or vegetative state; 6 points: Death.

### 2.3 Statistical analysis

Statistical analysis was performed using SPSS version 23.0. Normally distributed continuous variables were expressed as mean ± standard deviation (mean ± SD), and categorical variables were expressed as number (percentage). The skewed continuous variables were expressed as median and interquartile range [IQR, P25–P75]. Normally distributed data between groups were analyzed by two-sample t-test, and skewed data were analyzed by the Mann–Whitney *U* test. The categorical variables were tested with Fisher's exact test. The *p*-value < 0.05 was considered statistically significance.

## 3 Results

### 3.1 Basic characteristics

Nine children were diagnosed with ANE, five were male and four were female, ranging in age from 9 months to 14 years (mean: 5.7 ± 4.4 years). Five patients (56%) were aged <4 years. Most cases (8/9, 89%) occurred in winter or spring. Eight children (89%) had no prior history of genetic metabolic or immunodeficiency diseases; and 1 case (11%) had undergone aortic balloon valvuloplasty for aortic stenosis at the age of 1.5 months, with good postoperative recovery. The median interval from fever onset to seizure or altered consciousness was 19 h (IQR 9–24), based on caregiver reports at admission. No significant differences were noted between survival and non-survival groups in terms of sex, age, underlying diseases, time from fever to neurological symptoms, or season of onset (*P* > 0.05) (Table 1).

**Table 1:** Comparison of general data between Two groups of children.

Characteristics	Survival group (*n* = 4)	Non-survival group (*n* = 5)	Z-score	*P*-value
Sex (Male/Female, n)	3/1	2/3	—	0.142
Age [M (P25, P75), months]	68 (29,138)	68 (23,116)	0.04	0.968
Underlying Diseases [n (%)]	1 (25)	0 (0)	—	0.171
Time from Fever Onset to Seizure or Altered Consciousness [M (P25, P75), h]	17 (9,24)	22 (9,36）	−1	0.317
Winter/Spring	1/2	2/3	—	0.787

### 3.2 Clinical manifestations, multisystem involvement, and laboratory findings

At PICU admission, the most frequent symptoms were fever (8/9, 89%), including hyperpyrexia in 4 cases (44%), seizure (6/9, 67%), and altered consciousness (9/9, 100%). Multisystem involvement was evident, including shock (5/9, 56%), cough (3/9, 33%), and vomiting (1/9, 11%). Organ dysfunction was also prevalent: acute liver injury (8/9, 89%), coagulation abnormalities (8/9, 89%), acute kidney injury (4/9, 44%), thrombocytopenia (5/9, 56%), and acute myocardial injury (6/9, 67%). The incidence of acute myocardial injury was notably increased in the non-survival group compared to survivors (*P* < 0.05).

Coagulation parameters revealed elevated PT in 8/9 (89%) patients, ranging from 13.7 to 50.1 s (reference: 8.7–14.7 s), elevated APTT in 7/9 (78%) patients, ranging from 33.2 to 108.3 s (reference: 20–39.4 s). Fibrinogen levels were decreased (<200 mg/dl) in 6/9 (67%) patients and elevated (>200 mg/dl) in 3/9 (33%) patients (reference: 200–200 mg/dl). D-dimer was elevated (>243 μg/L) in most patients, with levels exceeding 500 μg/L and and some above 1,000 μg/L (reference: 0–243 μg/L). These findings suggest coagulation dysfunction most likely due to systemic inflammation, liver injury, or consumptive coagulopathy (Table 2).

**Table 2:** Comparison of clinical characteristics and laboratory test results between two groups of children.

Characteristics	Survival group (*n* = 4)	Non-survival group (*n* = 5)	Z-score	*P*-value
Clinical Characteristics [n (%)]				
Hyperpyrexia	1 (25)	3 (60)	—	0.254
Seizure	2 (50)	4 (80)	—	0.18
Altered consciousness	4 (100)	5 (100)	—	1
Shock	2 (50)	3 (60)	—	0.652
Vomiting or diarrhea	0 (0)	1 (20)	—	0.373
Cough	0 (0)	3 (60)	—	0.18
Acute liver injury	3 (75)	5 (100)	—	0.18
Coagulation Dysfunction	3 (75)	5 (100)	—	0.18
Acute kidney injury	1 (25)	3 (60)	—	0.117
Acute myocardial injury	1 (25)	5 (100)	—	0.034
Thrombocytopenia	2 (50)	3 (60)	—	0.652
Laboratory tests				
CRP[M (P25, P75), mg/L]	33.3 (7.6,111.3)	40 (8.9,63)	0.55	0.59
PCT[M (P25, P75), ng/ml]	53 (13,56)	62 (26,99)	−1.09	0.3
PLT[M (P25, P75), *10^9 ^/L]	119 (39,237)	99 (38,110)	1.33	0.22
Lac[M (P25, P75), mmol/L]	2.8 (1.2,9.9)	4.9 (3.9,6)	−0.19	0.85
ALT[M (P25, P75), U/L]	89 (16,9378)	161 (121,1730)	−0.98	0.327
AST[M (P25, P75), U/L]	182 (25,19678)	350 (275,2258)	−0.74	0.459
D-D[M (P25, P75), μg/L]	17,145	32,360 (17500,53170)	−0.49	0.624
IL-6[M (P25, P75), pg/ml]	2,937 (1092,6961)	576 (196,5896)	−1.23	0.218
IL-10[M (P25, P75), pg/ml]	838 (15,2431)	227 (166,823)	0	1.000
CSF Protein[M(P25, P75),mg/dl]	38 (7,318)	349 (99,450)	0.58	0.562

[CRP] C-reactive protein, reference range: 0–8 mg/L; [PCT] Procalcitonin, reference range: 0–0.05 ng/ml; [PLT] Platelets, reference range: 100–300 × 10⁹ /L; [Lac] Lactic acid, reference range: 0.7–2.1 mmol/L; [ALT] Alanine transaminase, reference range: 7–30 U/L; [AST] Aspartate transaminase, reference range: 14–44 U/L; [D-D] D-dimer, reference range: 0–243 μg/L; [IL-6] Interleukin-6, reference range: 0–16.6 pg/ml; [IL-10] Interleukin-10, reference range: 0–4.9 pg/ml; [CSF Protein] Cerebrospinal fluid protein, reference range: 20–40 mg/dl.

Acute myocardial injury was observed in 6 of 9 patients (67%), indicated by elevated cardiac troponin I (cTnI) levels above the 99th percentile upper reference limit (0.028 μmol/L). Levels were significantly higher in non-survivors (mean 0.99 μmol/L) than in survivors (mean 0.11 μmol/L), with overall values ranged from 0.022 to 4.35 μmol/L. Electrocardiographic abnormalities included ST segment depression, T wave changes, and conduction disturbances such as incomplete right bundle branch block. Echocardiography (ECG) abnormalities such as ST-segment depression and T wave inversion were observed in 4 of the 6 patients with elevated troponin, and all of whom were in the non-survival group. TTE revealed decreased left ventricular wall motion and a left ventricular ejection fraction (LVEF) as low as 26% in the non-survival group, while survivors exhibited normal cardiac function. Elevated B-type natriuretic peptide (BNP) levels, ranging up to as high as 4,170 pg/ml, indicated myocardial stress or dysfunction. Blood pressure measurements ranged from hypotension (the lowest 68/34 mmHg) in critical cases to normal or elevated levels in others.

The survival and non-survival groups presented with elevated inflammatory markers, including CRP, PCT, lactate, transaminases, D-dimer, IL-6, IL-10, and CSF protein (one case was unable to undergo CSF analysis due to severity). Platelets counts were mildly decreased without significant difference between groups (*P* > 0.05).

Respiratory pathogen testing was positive in 8/9 (89%), of which influenza A virus (5/9, 56%), influenza B virus (1/9, 11%), mycoplasma pneumoniae (1/9, 11%), and SARS-CoV-2 (1/9, 11%) were detected. The non-survival group had a significantly higher influenza A virus positivity than survival group (5/5 vs. 1/4, *P* = 0.022).

### 3.3 Neuroradiological findings

All nine children were cranially imaged upon admission. Five cases (Cases 1–5), critically ill or dying shortly after admission, had only cranial CT due to unstable clinical conditions, which precluded MRI. CT scans were done within 1–2 days of admission, with follow-up imaging in 3 cases. Case 1 showed normal findings and the remaining four patients exhibited scattered low-density on the images, which is suggestive of early cerebral involvement.

Among the four surviving patients (Cases 6–9), cranial MRI on admission was performed. Three patients (Cases 6–8) received serial MRI scans over intervals ranging from 5 days to 4 months post-admission, and Case 9 underwent a single MRI on day 5 post-admission. Serial MRIs were analyzed for Cases 6–8, which allowed for a more detailed evaluation of disease progression over time. The imaging findings were consistent with typical ANE patterns, showing bilateral, symmetrical, and multifocal lesions primarily involving the thalami, as well as the lentiform and caudate nuclei.

**Figure 1:**
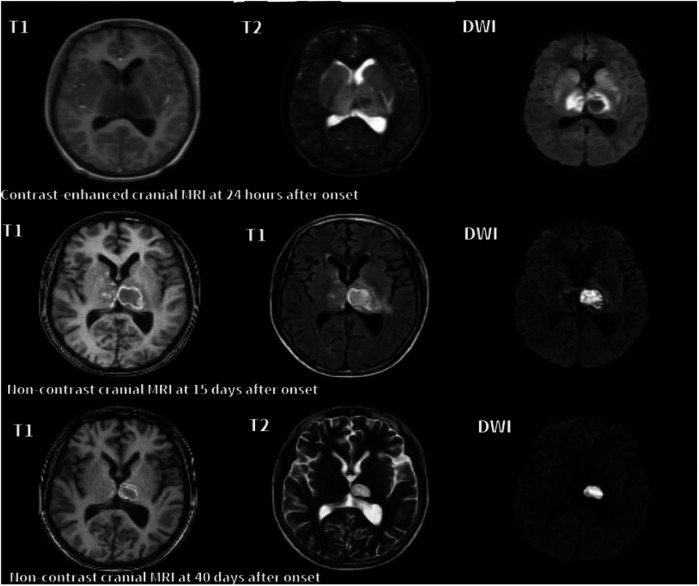
Serial axial MRI images of case 6 with ANE. **24 h after onset**: T1 shows mild swelling of the bilateral thalami, while T2 and DWI reveal symmetrical hyperintensities in the thalami, lentiform nuclei, and caudate nuclei, consistent with cytotoxic edema. Marked restricted diffusion is also observed in the thalami. **15 days after onset**: T1, T2, and DWI show reduced swelling with heterogeneous signal changes and evolving hemorrhagic transformation in the bilateral thalami and basal ganglia. Persistent restricted diffusion is noted in the right thalamus. **40 days after onset**: T1, T2, and DWI show partial resolution of lesions and volume loss in the thalami, indicative of cystic degeneration and encephalomalacia.

**Figure 2:**
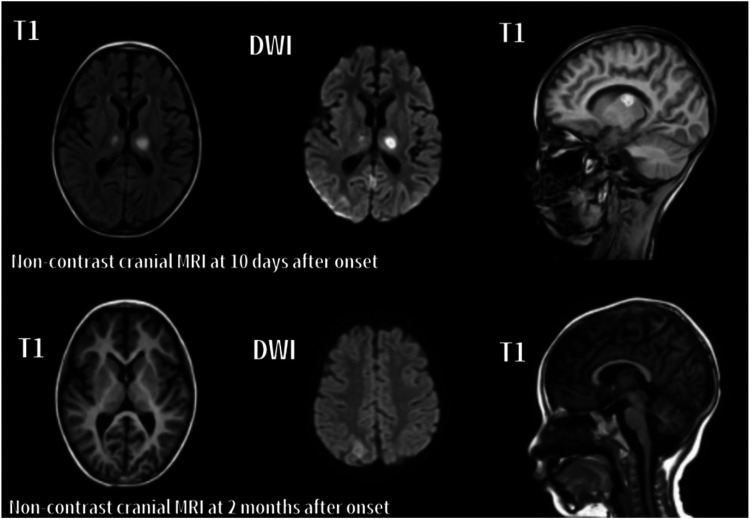
Serial axial and sagittal MRI images of case 7 with ANE. The left and middle columns display axial images, while the right column shows sagittal images. **10 days after onset**: Patchy abnormal signals are observed in the bilateral thalami, cerebellar hemispheres, and right parietal lobe. Lesions appear slightly hyperintense on T1, and hyperintense on T2 and DWI. **2 months after onset**: T1, T2, and DWI show reduced lesion size and signal intensity in the previously involved regions, indicating radiological resolution.

In the acute phase (<1 month), T2-weighted imaging (T2) and diffusion-weighted imaging (DWI) revealed symmetrical hyperintensities in the bilateral thalami and basal ganglia, consistent with cytotoxic edema and restricted diffusion. In some cases, T1-weighted imaging (T1) demonstrated hyperintense lesions with surrounding hypointense rims, forming a characteristic “concentric ring” sign. Serial MRI scans in Cases 6 and 7 illustrated these typical ANE patterns. In the recovery phase (>2 months), serial MRIs in both cases showed partial or complete resolution of lesions. Notably, Case 6 exhibited cystic degeneration of the thalami, indicative of encephalomalacia. Both patients are currently undergoing rehabilitation (Figures 1, 2).

### 3.4 Disease severity and treatment

ANE-SS was significantly lower in the survival group than in the non-survival group (*P* < 0.05). High-risk ANE-SS scores (5–9 points) were more frequently observed in the non-survival group. Risk stratification was defined as follows: 0–1 points = low risk, 2–4 points = moderate risk, 5–9 points = high risk.

Antiviral therapy was administered to 6 of 9 patients (67%), and the most commonly used agent was oseltamivir. Four patients (Cases 4, 6, 8, and 9) received oseltamivir on the day of admission. Oseltamivir was given orally using a standard pediatric dose of 3 mg/kg twice daily, age and weight adjusted,with therapy lasting from 5 to 8 days. Case 3 was given a single oral dose of baloxavir marboxil on admission day 1. Acyclovir was administered for two days in Case 2, who unfortunately died at an early stage during hospitalization. Three patients (Cases 1, 5, and 7) did not receive antiviral therapy. Antiviral regimens were selected based on respiratory pathogen identification and clinical judgment.

All patients (9/9) received high-dose intravenous methylprednisolone (≥10 mg/kg/day) of which four received 10 mg/kg/day, another four received 20 mg/kg/day, and one patient received 30 mg/kg/day. There were no statistically significant differences in methylprednisolone dosing between survival and non-survival groups.

Eight of nine patients (89%) received immunomodulatory therapy with intravenous immunoglobulin (IVIG) or/and plasma exchange (PLEX), with increased usage observed in the non-survival group. Invasive mechanical ventilation (IMV) was required for all patients in the non-survival group, initiated immediately after PICU admission. There were no significant differences between groups regarding IMV duration or PICU length of stay (*P* > 0.05) (Table 3).

**Table 3:** Comparing of treatment and prognosis-related indicators between Two groups of children admitted to PICU.

Characteristics	Survival group (*n* = 4)	Non-survival group (*n* = 5)	Z-score	*P*-value
ANE-SS [Score, M (P25, P75)]	4.5 (3.5,5)	8 (6,8)	−4.64	<0.0001
ANE-SS High Risk [*n* (%)]	2 (50)	5 (100)	—	0.167
Antiviral Drugs [*n* (%)]	3 (75)	3 (60)	—	0.595
Methylprednisolone [*n* (%)]	4 (100)	5 (100)	—	1
IVIG [*n* (%)]	3 (75)	5 (100)	—	1
PLEX [*n* (%)]	3 (75)	5 (100)	—	1
Invasive Mechanical Ventilation [*n* (%)]	3 (75)	5 (100)	—	1
Invasive Mechanical Ventilation Duration [d, M (P25, P75)]	6.5 (2,10)	13 (10,16)	−1.7	0.09
Length of PICU Stay [d, M (P25, P75)]	25.5 (16,28)	13 (10,16)	1.93	0.09

IVIG, intravenous immunoglobulin; PLEX, plasma exchange; PICU, Pediatric Intensive Care Unit.

### 3.5 Outcome and follow-up

Five of 9 cases died. One of the patients succumbed to multi-organ failure within 24 h of admission. Two patients died post-discharge due to severe encephalopathy, long-term dependence on IMV, and failure to wean from respiratory support. The remaining two patients died 10–15 days after admission due to cerebral edema (leading to brain herniation and loss of brainstem reflexes) and circulatory failure. Four survivors received post-discharge rehabilitation therapy and demonstrated significant improvements in consciousness, motor function, and intelligence compared to discharge. Follow-up assessments, conducted via telephone at 1, 3, and 6 months post-discharge, revealed that one child achieved normal somatic growth (height and weight) and immune function. Two children experienced chronic motor impairment, while one exhibited severe neurological sequelae including urinary and fecal incontinence, motor dysfunction, and fluctuating consciousness, but without seizures (Table 4).

**Table 4:** Follow-up outcomes of survivors with acute necrotizing encephalopathy.

Survivor children	1-month POPC score	3-month POPC score	6-month POPC score
NO.1	2	1	1
NO.2	4	3	3
NO.3	3	2	2
NO.4	3	2	2

POPC, pediatric overall performance category.

## 4 Discussion

We retrospectively reviewed nine children diagnosed with ANE admitted to the PICU of the Women's and Children's Hospital of Ningbo University between March 2,019 and September 2024. Six cases (67%) were less than 6 years old (range 9 months to 14 years). Most cases (89%) occurred in winter or spring, consistent with previous seasonal trends (9). Influenza A/B viruses were the most common pathogens, followed by *Mycoplasma pneumoniae*, and SARS-CoV-2. The non-survivor group had significantly higher influenza positivity, as in previous studies that found influenza in 34.6%–80% of ANE cases (6 10–11).

At the time of PICU admission, fever (89%), hyperpyrexia (≥40°C, 44%), seizures (67%), and altered consciousness (100%) were common, with shock present in 56%. Hyperpyrexia can reflect an aggressive inflammatory response and has been linked to worse neurological outcome in ANE (12). Therefore, we recorded both fever and hyperpyrexia separately to examine their potential prognostic significance. Shock was a significant mortality predictor, with previous research showing near 100% mortality and a 14 times fold increased risk of death (11, 13). In this research, the non-survivor group witnessed more shock events than survivors (60% vs. 50%), consistent with previous research. All the patients presented with markers of inflammatory and tissue injury at PICU admission, especially IL-6. This agrees with the established ANE pathogenesis of an immune response-induced dysregulated cytokine storm (1, 14). The subsequent systemic manifestations are liver and kidney injury, shock, and disseminated intravascular coagulation. In addition, disruption of the blood-brain barrier leads rise to multifocal brain lesions and increased CSF protein (15, 16).

Neuroimaging is of utmost significance in the early diagnosis of pediatric ANE, and symmetrical bilateral gray and white matter involvement, particularly in the bilateral thalami, basal ganglia, periventricular areas, and centrum semiovale, is a typical feature (17). Radiological changes evolve dynamically, reflecting a transition from edema to petechiae, hemorrhage, and eventually necrosis (18, 19). The acute phase, a typical “tricolor plate pattern” can be observed on DWI and apparent diffusion coefficient (ADC) maps, characterized by a central hyperintense core surrounded by hypointense rings, indicative of vasogenic edema. Perithalamic hyperintensity on DWI represents cytotoxic edema, while Hemorrhagic foci appear on susceptibility-weighted imaging (SWI). Three-layered appearance varies with time of imaging and disease severity. Serial MRI tends to demonstrate encephalomalacia or secondary brain atrophy (17–19). Imaging within our cohort was consistent with these typical patterns.

Prognostically, ANE-SS scores were lower in survivors, whereas the non-survivor group had a significantly higher proportion of high-risk scores. The finding is consistent with Li et al., who identified a 4-fold increased risk of mortality in patients with high ANE-SS when compared to those with moderate or low-risk scores (11). First reported by Yamamoto et al. (7), the ANE-SS has been validated as a strong predictor of mortality and long-term neurological sequelae. Validity may be compromised in the critically ill patients with limitation in the procurement of cranial MRI scans in extreme hemodynamic instability, while CT is not sensitive for brainstem lesion detection. Therefore, larger prospective studies are warranted to further refine prognostic score systems and early reliable predictors of clinical outcome. However, there remains no consensus on standardized immunosuppressive regimens in the management of ANE. The treatment modalities used presently mainly include high-dose methylprednisolone, IVIG, PLEX, and targeted biologics such as tocilizumab (1). Nevertheless, the efficacy, dosing, timing, combinations, and effectiveness of these modalities remain in controversy (20).

High-dose methylprednisolone is usually recommended by earlier studies, ideally within 12–24 h after the onset of neurological symptom. Doses of ≥20 mg/kg/day were associated with improved survival and neurological recovery (18, 21, 22). However, no universally accepted regimen or treatment duration currently exists. IVIG is frequently used in clinical practice, but its effectiveness is questionable. Meta-analyses and individual studies show no additional survival benefit of IVIG compared to corticosteroids, either as monotherapy or in combination (20, 23). The lack of randomized controlled trials only further complicates its clinical application and consensus on timing, dose, and selection of patients more challenging. PLEX has also been utilized more commonly as an adjunctive therapy. Current recommendations recommend 1–3 sessions, each replacing 1–1.5 times the patient's plasma volume, repeated every 24–48 h to remove cytokines (24). However, uniform protocols for timing of initiation and patient selection are still lacking, limiting its overall clinical use.

Tocilizumab, a monoclonal antibody targeting the IL-6 receptor, has also been associated with favorable outcomes in recent case series and observational reports. Early administration appears to lower cytokine storm and is associated with good long-term neurological outcomes (25–27). To tackle pressing concerns, future large-scale, multicenter randomized controlled trials are a high priority. These studies should compare immunomodulatory regimens systematically, optimize the treatment windows, and correlate biomarker-guided, personalized treatment strategies (28, 29).

Our findings also highlight the prognostic significance of hyperpyrexia as an early clinical indicator, often underappreciated, and confirm the association of influenza infection with adverse outcomes, stressing the need for heightened clinical vigilance. The mortality rate of our group was 56% (5/9), with the only one survivor (11%) having full neurological recovery on six-month follow-up, consistent with previous reports (5, 6). These results underscore the importance of early diagnosis and personalized immunomodulatory therapies based on disease severity and etiology.

There are some limitations to this study. First, there are few patients and one-site design that impose limitations on statistical power and generalizability. Second, selection bias was possible because a few patients (mostly patients of early death) were not scanned with cranial MRI, which may lead to underestimation of radiological spectrum of ANE. Initially, cranial CT was used, although MRI is of higher resolution, particularly for early and recovery phases. All four survivors also underwent baseline MRI. Three of them (Cases 6–8) also underwent serial scans from 5 days to 4 months, whereas Case 9 underwent an isolated MRI, limiting radiological progression assessment. Third, telephone follow-up was utilized primarily. Although three survivors underwent outpatient examination with serial MRI, long-term neurological outcome lacked objective and complete assessment since in-person standardized neurological examination was not available. Fourth, the relatively short and heterogeneous follow-up period lead to inadequate prognostic assessment. Fifth, unaccountable confounders such as genetic determinants, co-infections, and treatment heterogeneity were poorly controlled, preventing causal inference of treatment effects. Large multicenter studies with uniform imaging protocol, long-term follow-up, and full neurological evaluation are needed to validate and extend these findings.

## 5 Conclusion

This retrospective study comprehensively examined the clinical, laboratory, imaging, and prognostic features of nine cases of childhood ANE in a systematic manner. The most common presenting symptoms were fever, altered consciousness, and seizure, and shock was an important risk factor for mortality. Elevated inflammatory markers, in particular IL-6, and cardiac and liver impairment, were common. Antecedent infections were predominantly influenza-based, as would be expected with seasonal viral seasonality. Cranial MRI mostly revealed characteristic symmetric “tricolor pattern” in bilateral thalami. Most patients were treated with a regimen of immunomodulatory therapies including high-dose methylprednisolone, IVIG, PLEX and tocilizumab. Although stratified comparisons between early and late interventions could not be made due to the retrospective design, the study suggests the possible prognostic implication of hyperpyrexia and highlights the importance of timely intervention. These findings serve as the basis for future planned prospective multicenter trials to compare systematically treatment regimens, dose, and timing, with the long-term aim of achieving optimum clinical outcomes in children with ANE. Future studies should clarify the role of early intervention and provide direct evidence to guide clinical practice.

## Data Availability

The raw data supporting the conclusions of this article will be made available by the authors, without undue reservation.
